# Molecular Drivers of Regional and Cellular Vulnerability to Tau Pathology and Neurodegeneration

**DOI:** 10.1002/alz70855_103209

**Published:** 2025-12-23

**Authors:** Diede Broekaart, Abhijeet Sharma, Aarthi Ramakrishnan, Saraswathi Subramaniyan, Minghui Wang, Vishwendra Patel, Bin Zhang, Lea T. Grinberg, Robert Blitzer, Eric Schmidt, Li Shen, Patrick R Hof, Ana C Pereira

**Affiliations:** ^1^ Icahn School of Medicine at Mount Sinai, New York, NY, USA; ^2^ UCSF Weill Institute for Neurosciences, University of California, San Francisco, CA, USA; ^3^ Rockefeller University, New York, NY, USA

## Abstract

**Background:**

Tauopathies are characterized by the aggregation and accumulation of hyperphosphorylated tau proteins that correlates with cognitive impairment in affected individuals. The presence of tauopathy follows a temporospatial spreading pattern in which certain neuronal cell types in specific brain regions are more vulnerable to tau accumulation and atrophy. However, the mechanisms underlying the selective vulnerability of these neurons and regions to pathological tau accumulation are not fully understood.

**Method:**

Post‐mortem human brain tissue of tauopathies was used to examine tau pathology in excitatory and inhibitory neurons through immunohistochemistry. For molecular studies, viral translating ribosome affinity purification was performed: GAD2‐ires‐cre and vGLUT1‐ires‐cre mice were bred with either wild‐type C57BL6/J (WT) or P301S (PS19) mice. Seven‐month‐old male mice were stereotaxically injected with AAV9.EF1a.Flex.eGFPL10a.WPRE.hGH in vulnerable and resistant brain regions to tau. TRAP‐translating‐ribosome‐affinity‐purification‐RNA‐sequencing was performed. Bioinformatics including Gene Ontology and Multiscale Embedded Gene Co‐Expression Network Analysis were performed. Further, mice were electrophysiologically‐characterized.

**Result:**

We characterized the presence of phosphorylated tau in excitatory and inhibitory neurons in post‐mortem‐prefrontal cortex of tauopathy, including Alzheimer's disease, progressive supranuclear palsy, corticobasal degeneration and frontotemporal lobar dementia‐MAPT. We observed that neuronal tau accumulation across these tauopathies occurs predominantly in excitatory neurons compared to inhibitory neurons. Next, we performed viral translating ribosome affinity purification(vTRAP) from vulnerable and resistant brain regions on vGLUT1^CRE+^ and GAD2^CRE+^ PS19 mice to understand molecular signatures of tau vulnerability. We observed that both vulnerable regions and neurons are characterized by alterations in synaptic transmission and neuronal excitability. Transcription factor Mef2c was identified as an upstream regulator affecting myelination and synaptic organization in vulnerable brain regions in PS19 mice. The relevance of these findings was validated in human tauopathies via coexpression network analysis. Concordantly, we observed tau‐induced reduction of excitatory and inhibitory synaptic density and interregional changes in spontaneous postsynaptic currents of excitatory neurons in mice.

**Conclusion:**

We conclude that excitatory neurons are most vulnerable to tau pathology across tauopathies and selective vulnerability to tau pathology could arise from changes in neurotransmission and synaptic compositions, potentially due to an altered Mef2c transcription network. This study provides important data on molecular drivers of regional and cellular susceptibility to neurodegeneration.

